# High-Efficiency Flexible Organic Photovoltaics and Thermoelectricities Based on Thionyl Chloride Treated PEDOT:PSS Electrodes

**DOI:** 10.3389/fchem.2021.807538

**Published:** 2022-03-01

**Authors:** Juanyong Wan, Xi Fan, Yunfei Li, Pengcheng Li, Ting Zhang, Kwun Nam Hui, Huihui Huang, Kai Kang, Lei Qian

**Affiliations:** ^1^ Division of Functional Materials and Nanodevices, Ningbo Institute of Materials Technology and Engineering, Chinese Academy of Sciences, Ningbo, China; ^2^ School of Physics and Electronics, Hunan University, Changsha, China; ^3^ Hubei Key Laboratory of Plasma Chemistry and Advanced Materials, Hubei Engineering Technology Research Center of Optoelectronic and New Energy Materials, School of Materials Science and Engineering, Wuhan Institute of Technology, Wuhan, China; ^4^ Joint Key Laboratory of the Ministry of Education, Institute of Applied Physics and Materials Engineering, University of Macau, Taipa, Macau SAR, China

**Keywords:** flexible transparent electrode, organic solar cell, thermoelectricity, PEDOT:PSS, conducting polymer

## Abstract

Conducting polymers have received tremendous attentions owing to their great potentials to harvest both luminous and thermal energies. Here, we reported a flexible transparent electrode of poly(3,4-ethylenedioxythiophene):poly(styrenesulfonate) (PEDOT:PSS) with highly electrical conductivity and raised Seebeck coefficient *via* thionyl chloride treatments. The comprehensive studies of optical, electrical, morphological, structural, and thermoelectrical properties, work function, and stability of the PEDOT:PSS transparent electrodes were systematically evaluated and described. On the basis of the PEDOT:PSS transparent electrodes, the resultant flexible organic solar cells yielded a high power conversion efficiency of 15.12%; meanwhile, the flexible thermoelectricities exhibited the raised power factor of 115.9 μW m^−1^ K^−2^, which outperformed the four kinds of rigid thermoelectricities with conventional acid and base treatments.

## Introduction

Developments of energy harvesting electronics such as flexible organic solar cells (OSCs) (Sun et al., 2019; Qu et al., 2020; Yao et al., 2020; Chen et al., 2020) and thermoelectricities (TEs) (Park et al., 2014; Xiong et al., 2015; Fan et al., 2017; Li et al., 2019; Jiang et al., 2020) have attracted a great deal of attentions recently. Flexible OSCs, which convert the luminous energy to electricity, have the wearable, portable, and light-weight advantages for a promising application into self-powered smart electronics; whereas flexible TEs, which convert the waste thermal energy to electricity, can be attached to surfaces dissipating heats for electricity generation. As a key device component, the flexible transparent electrode and thermoelectric layer of poly(3,4-ethylenedioxythiophene):poly(styrenesulfonate) (PEDOT:PSS) have the striking virtues, such as aqueous solution manufacturing, low cost, high conductivity (*σ*), adjustable work function, and good thermal stability, thereby enabling a promising adaptation of them into flexible OSCs and flexible TEs (Na et al., 2008; Kim et al., 2011; Xia et al., 2012; Kim et al., 2014; Fan et al., 2015; Meng et al., 2015; Worfolk et al., 2015; Song et al., 2018; Fan et al., 2019; Fan, 2021; Wan et al., 2020a; Wan et al., 2020b; Wen et al., 2021; Wan et al., 2021).

As the most commercial conducting polymers (CPs), PEDOT:PSS (Clevios PH1000) transparent electrodes exhibited a highly electrical conductivity over 3,000 S cm^−1^ *via* strong acid treatments (Xia et al., 2012; Fan et al., 2015). The electrical conductivity was substantially improved after 1) diluted strong acid treatments at high temperatures and 2) fully concentrated strong acid treatments. However, the PEDOT:PSS transparent electrodes coated on plastic substrates suffered from these drawbacks, including inhomogeneous morphology with high roughness, large-domain aggregates with sizes of 30–50 nm in matrices, and limited flexibility (Fan et al., 2015; Fan et al., 2020; Wan et al., 2020a). These factors presumably restrain the power conversion efficiency (PCE) of indium tin oxide (ITO)–free flexible OSCs.

PEDOT:PSS has been also employed as thermoelectric materials (Park et al., 2014; Xiong et al., 2015; Fan et al., 2017; Li et al., 2019; Xu et al., 2021). The power factor (PF) of TEs is defined as PF = *S* ^2^ *σ*, where *S* is the Seebeck coefficient (Tritt and Subramanian, 2006; Snyder and Toberer, 2008). To improve the PF of the PEDOT:PSS electrodes, strong acid treatments were commonly employed; however, the strong acid approach commonly result in a rather low PF no more than 58.7 μW m^−1^ K^−2^. To improve the thermoelectric performance of the PEDOT:PSS electrodes, a few methods including 1) secondary polar solvent and base multistep treatments, 2) acid and base multistep treatments, and 3) hybrid strategies had been subsequently developed. It should be mentioning that the base treatments employed not only made the processing procedure more complex but also led to a deeply blue PEDOT:PSS thin film, thereby unsuitable to make transparent PEDOT:PSS electrodes.

**FIGURE 1:**
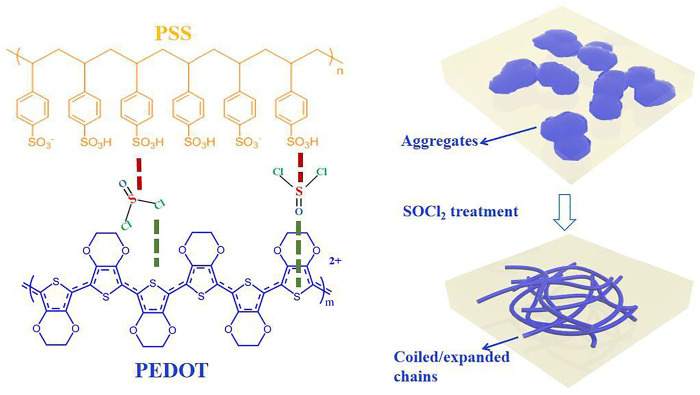
Schematic diagrams of inter-molecular interactions among PEDOT, PSS, and SOCl_2_, and morphology evolution of the PEDOT:PSS films induced by SOCl_2_.

Thionyl chloride (SOCl_2_) molecules involve rich sulfur–chlorine (S–Cl) and sulfur = oxygen (S=O) polar covalent bonds. The polar SOCl_2_ molecules potentially reduce the Coulomb attractions between PEDOT and PSS for a highly electrical conductivity (see inter-molecular interactions in Figure 1). The polar covalent bonds also polarize the conducting PEDOTs for raising the work function (*Φ*) of the PEDOT:PSS films. A high *Φ* minimizes the energy level mismatch among the PEDOT:PSS anodes, hole transporting layers (HTLs), and electron donors of photo-active layer of OSCs for an efficient charge transport. Therefore, the SOCl_2_-treated PEDOT:PSS electrodes maybe suitable for flexible ITO-free OSCs and TEs.

Herein, we propose the SOCl_2_ treatment to energetically raise the electrical conductivity, Seebeck coefficient, and work function of the PEDOT:PSS electrodes. The SOCl_2_ treatment did lead to a smooth, uniform, and homogeneous PEDOT:PSS electrode that consisted of expanded/coiled chains (see morphology evolution in Figure 1). The electrical conductivity and work function was raised to 2,230 S cm^−1^ and 4.89 eV, respectively. The solution-processed flexible OSCs yielded a considerably high PCE of 15.12%, which was comparable to that of the rigid OSCs with 110-nm-thick ITO-coated glass substrates. Meanwhile, the flexible TEs exhibited a high Seebeck coefficient of 22.8 μV K^−1^ and PF of 115.9 μW m^−1^ K^−2^ with highly optical transmittance. The work demonstrates the synergistic enhancements in σ, *S*, and *Φ* of PEDOT:PSS electrodes by SOCl_2_ and the promising applications into flexible energy harvesting electronics.

## Experimental

### Materials

PM6 and Y6 were purchased from Solarmer Materials Inc., Beijing. PEDOT:PSS aqueous solutions (Clevios PH1000 and P VP AI4083) were purchased from Heraeus, Germany.

### Solar Cell Fabrication

For the rigid ITO-based solar cells, glass substrates (size: 2.0 cm × 2.0 cm) were cleaned through using ultrasonic treatments in deionized (DI) water, acetone, and isopropyl alcohol (IPA) and then were processed in UV-ozone chambers for 10 min. For the flexible solar cells, the polyethylene terephthalate (PET) plastic substrates were cleaned by IPA followed by baking at 100°C for 10 min. Next, PEDOT:PSS aqueous solutions (PH1000) were filtered through 0.45-µm syringe filter. The PEDOT:PSS (PH1000) solutions were spin-coated on underlying substrates at 3,500 rpm, and the as-cast PEDOT:PSS films were dried at 80°C for 15 min in the air atmosphere. The spin-coating deposition of PEDOT:PSS (PH1000) and the drying treatment at 80°C were conducted again. Then, the SOCl_2_ soaking treatment was conducted *via* dipping the >99 wt.% SOCl_2_ solutions on the PEDOT:PSS surfaces at room temperature. Upon the SOCl_2_ treatment for 10 min, the SOCl_2_ residues were washed off by DI water and IPA followed by baking at 80°C for 15 min.

Subsequently, a PEDOT:PSS HTL (Clevios P VP AI4083) was spin-coated on the ITO and PEDOT:PSS electrodes at 3,000 rpm for 30 s, and the HTL was dried at 100°C for 10 min. After that, the photo-active layer blends of PM6:Y6 were dissolved in chloroform with 1-chloronaphthalene (0.5%, v/v) and were spin-coated at 3,000 rpm for 30 s on the PEDOT:PSS (P VP AI4083) HTL. Then, perylene diimide amino N-oxide (PDINO) in methanol (1.0 mg ml^−1^) were spin-coated on the surfaces of photo-active layers at 2,000 rpm to obtain the electron transporting layers. Finally, the cathodes of Al were thermally evaporated under a pressure at 10^−4^ Pa. The active area is 4.00 mm^2^. Note that metal probes were contacted with Al top cathodes and the PEDOT:PSS bottom anodes coated with silver (Ag) pastes for the current density–voltage (*J−V*) characteristics.

### Thermoelectricity Fabrication

PEDOT:PSS aqueous solutions (Clevios PH1000) were filtered through 0.45-µm syringe filter. The PH1000 solutions were spin-coated on the clean PET substrates at 3,500 rpm followed by the drying at 80°C for 15 min in the air atmosphere. The spin coating of PH1000 and the drying treatment were conducted again. Subsequently, the sulfuric acid (H_2_SO_4_) and methanesulfonic acid (CH_3_SO_3_H) treatments were conducted *via* dipping these acid solutions on the PEDOT:PSS surfaces with a controlled temperature of 140°C or room temperature, respectively; whereas the SOCl_2_ treatment was conducted *via* dipping the SOCl_2_ solution on the PEDOT:PSS surfaces at room temperature. Upon the treatments for 10 min, the acid and SOCl_2_ residues were washed off by DI water and IPA followed by baking at 80°C for 15 min. In the following base treatment, 0.1 M NaOH aqueous solutions (100 μl) were dropped onto the acid-treated PEDOT:PSS films. The NaOH base treatment was conducted for 1 min at room temperature. The as-treated films were rinsed with DI water for three times followed by drying on a hot plate at 100°C for 5 min. Finally, two Ag line electrodes with a length of 1.5 cm and a distance of 2 mm were prepared *via* using Ag pastes.

### Characterizations

Sheet resistance was measured through using the van der Pauw method. Film thickness was conducted by a surface profile meter (TalySurf Series II). UV–vis spectra were taken on GS54T spectrophotometers (Shanghai Lengguang Technology Co., China). Film morphology was conducted using scanning probe microscope (SPM, VEECO Dimension 3100V). Element compositions of the PEDOT:PSS films were conducted by X-ray photoelectron spectroscopy (XPS, XSAM800). UV photoelectron spectra (UPS) measurements were carried out using a Kratos AXIS ULTRA DALD UPS system. The *J−V* characteristics were measured in the nitrogen (N_2_)–filled glove boxes using a Keithley 2400 sourceMeter under the illumination of AM 1.5G, with a AAA solar simulator (Newport, model 94023A). The lamp was calibrated by a 2 cm × 2 cm monocrystalline silicon reference cell (KG5 filter) provided by Newport Corporation. The light intensity was calibrated with a standard silicon detector. External quantum efficiency (EQE) was conducted through the Newport quantum efficiency measurement system (ORIEL IQE 200TM) in the ambient atmosphere. The light intensity was calibrated with a standard Si/Ge solar cell.

The Seebeck coefficient was measured by using a home-built system as previously reported in the previous literature 10. One side of the film was heated up by the Peltier device as hot end, whereas the other side was maintained in atmosphere as cold end. The temperature gradient (Δ*T*) was measured by *T*-type thermocouples and varied Δ*T* could be generated by controlling the applied power for the Peltier device. Thermoelectric voltage (Δ*V*) was obtained by using a Keithley 2000 multimeter, and the slope of the linear fitting of Δ*V*–Δ*T* plots was calculated to be the Seebeck coefficient.

## Results and Discussion

### Opt-Electrical Characteristics of PEDOT:PSS Films

**FIGURE 2:**
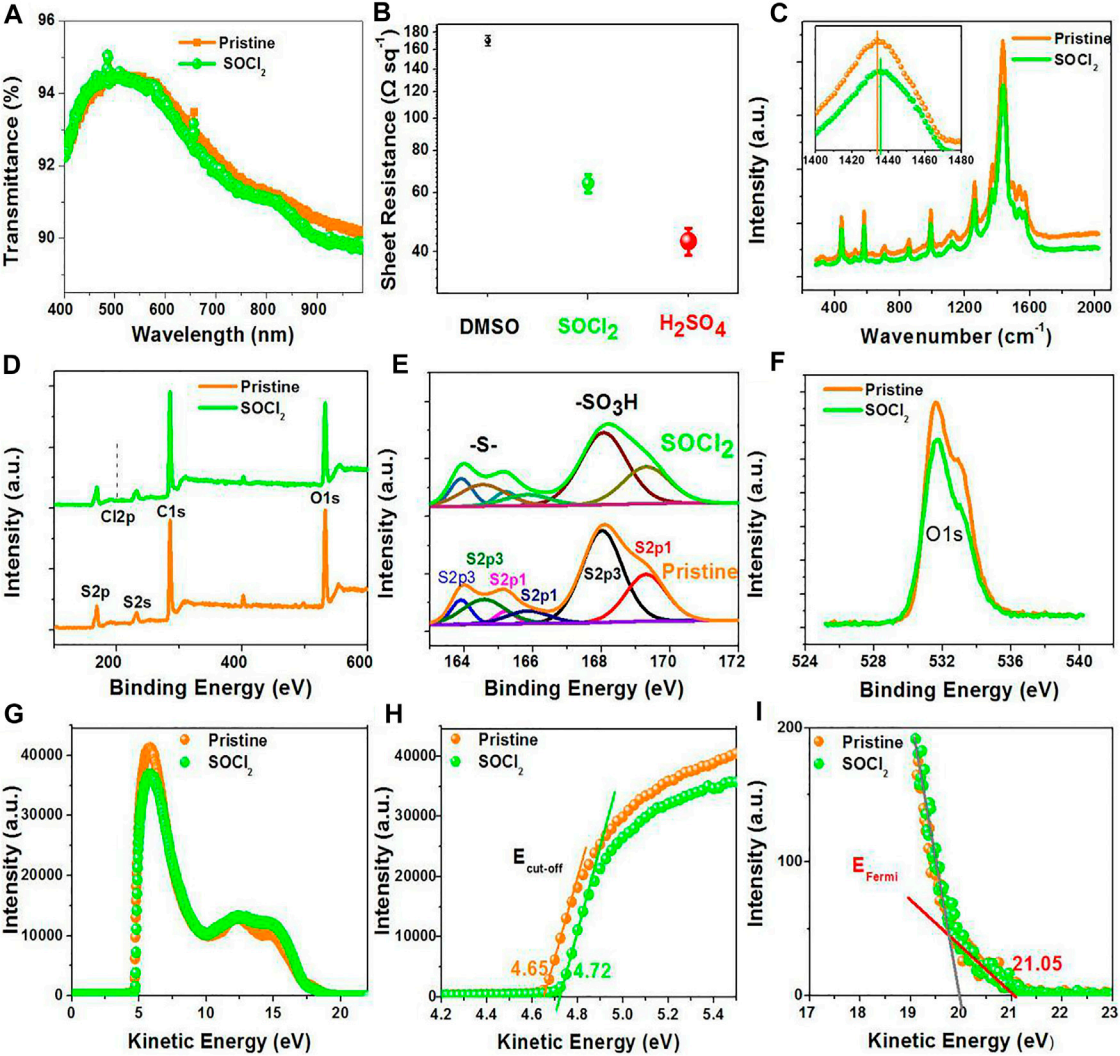
**(A)** Transmittance spectra of the pristine and SOCl_2_-treated PEDOT:PSS films. **(B)** Sheet resistance of the PEDOT:PSS films with 6 vol.% DMSO treatment, >99 wt.% SOCl_2_ treatment, and 98 wt.% H_2_SO_4_ treatment. **(C)** Raman, **(D)** Total XPS, **(E)** S2p XPS, **(F)** C1s XPS, **(G)** UPS, **(H)** E_cutoff_ region, and **(I)** E_Fermi_ region spectra of the PEDOT:PSS films.

The fundamental investigations of the PEDOT:PSS thin films with SOCl_2_ treatments are systematically introduced. The SOCl_2_ approach boosts the electrical conductivity, work function, Seebeck coefficient as well as stability, and directly links to the underlying mechanism of the enhanced PCE for flexible OSCs and the improved PF for flexible TEs. Figures 2A,B show the optical transparency (*T*%) and sheet resistance (*R*_sh_) of the pristine PEDOT:PSS thin film and the PEDOT:PSS electrodes (70-nm thickness) with the SOCl_2_ treatment at room temperature, respectively. After the SOCl_2_ treatment, *T*% was slightly decreased from 94.4 to 94.3% at λ = 550 nm and from 90.6 to 90.0% at λ = 900 nm, which were accompanied with a morphology evolution of the PEDOT:PSS electrodes. A high optical transmittance was generally achieved *via* improving the oxidation levels of PEDOT molecules and/or largely reducing the insulating and hydrophilic PSS components (Khan et al., 2015; Reyes-Reyes and Lopez-Sandoval, 2018). Here, the transmittance results suggest no visible raise of the oxidation levels of the PEDOTs by the SOCl_2_ treatment. Upon the SOCl_2_ treatment, the PEDOT:PSS electrodes showed a *R*_sh_ of 64 Ω sq^−1^, which was much lower than that (170 Ω sq^−1^) of the conventional PEDOT:PSS ones with 6 vol.% dimethyl sulfoxide (DMSO) treatments and higher than that (43 Ω sq^−1^) of the PEDOT:PSS ones with 98 wt.% H_2_SO_4_ treatments. Note that 98 wt.% H_2_SO_4_ treatments destroyed plastic underlying substrates, thereby unsuitable to prepare flexible PEDOT:PSS electrodes.

### Structure, Composition and Work Function of PEDOT:PSS Films

Figure 2C shows the Raman spectra of the PEDOT:PSS films, including the pristine ones and SOCl_2_-treated ones. The strongest band between 1,400 and 1,500 cm^−1^ originated from the C_α_ = C_β_ stretching vibration of the PEDOTs (Garreau et al., 1999). The C_α_ = C_β_ vibration peak in Raman spectroscopy blue-shifted only 1.6 cm^−1^. We found that this result was different from strong acid-treated PEDOT:PSS electrodes that exhibited a red-shift phenomenon. The result indicates a slight/rather limited change of the PEDOTs from quinoid structures to benzoid structures. The peak at 700 cm^−1^ corresponds to the sym C−S−C vibrations in PEDOTs. Both peaks had a similar intensity and half-height width, thereby supporting the conclusion that there was no visible structure evolution of the PEDOTs.

To elucidate the effects of the SOCl_2_ treatment on the components of the PEDOT:PSS films, XPS spectra were conducted (see Figures 2D–F). The binding energy at 201 eV corresponds to the Cl2p peak. As shown in Figure 2D, the ratio of Cl in SOCl_2_ to total atoms is no more than 0.15 atom.% for the SOCl_2_-treated films, suggesting little SOCl_2_ residuals on PEDOT:PSS surfaces; whereas a conventional treatment using high-concentration acids caused a large residual of acids that would affect the OSC efficiency. For example, the acid residual value was as high as 14.08 atom.% for the PEDOT:PSS electrodes with 85 wt.% phosphoric acid (H_3_PO_4_) treatment, and as-packaged flexible OSCs with the H_3_PO_4_-treated PEDOT:PSS electrodes showed a sharp decay of 17% when exposed in ambient air for 30 days (Fan et al., 2016).

For the pristine PEDOT:PSS films, the ratio of sulfur (S) atoms in sulfonate moieties of PSS to S atoms in thiophene rings of PEDOT (called S_PSS_:S_PEDOT_) is 2.42:1; whereas, for the SOCl_2_-treated films, the ratio is decreased to 2.07:1 (see Figure 2E), indicating a small removal of hydrophilic PSS from the PEDOT:PSS matrices. It ensures an intimate contact between the SOCl_2_-treated PEDOT:PSS electrodes and PEDOT:PSS (Clevios P VP AI4083) HTLs. Besides, the content of the oxygen (O) atoms is decreased from 27.4 to 24.1 atom.% after the SOCl_2_ treatment (see Figure 2F), supporting the removals of the hydrophilic PSS components.

The energy levels of the PEDOT:PSS thin films were probed as well through UV photoelectron spectra, as shown in Figure 2G. Figures 2H,I show the low kinetic energy cutoff (E_cutoff_) and the Fermi levels (E_Fermi_) of the pristine- and SOCl_2_-treated PEDOT:PSS films. The SOCl_2_-treated films exhibited a work function of 4.89 eV that was higher than that (4.82 eV) of the pristine ones. The higher work function was probably attributed to the strong electron-withdrawing Cl groups of the SOCl_2_ molecules. It allows a formation of Ohmic contacts and is favored for hole transport from photo-active layers of normal OSCs to the PEDOT:PSS electrodes treated by SOCl_2_.

### Morphology and Stability of PEDOT:PSS Films

**FIGURE 3:**
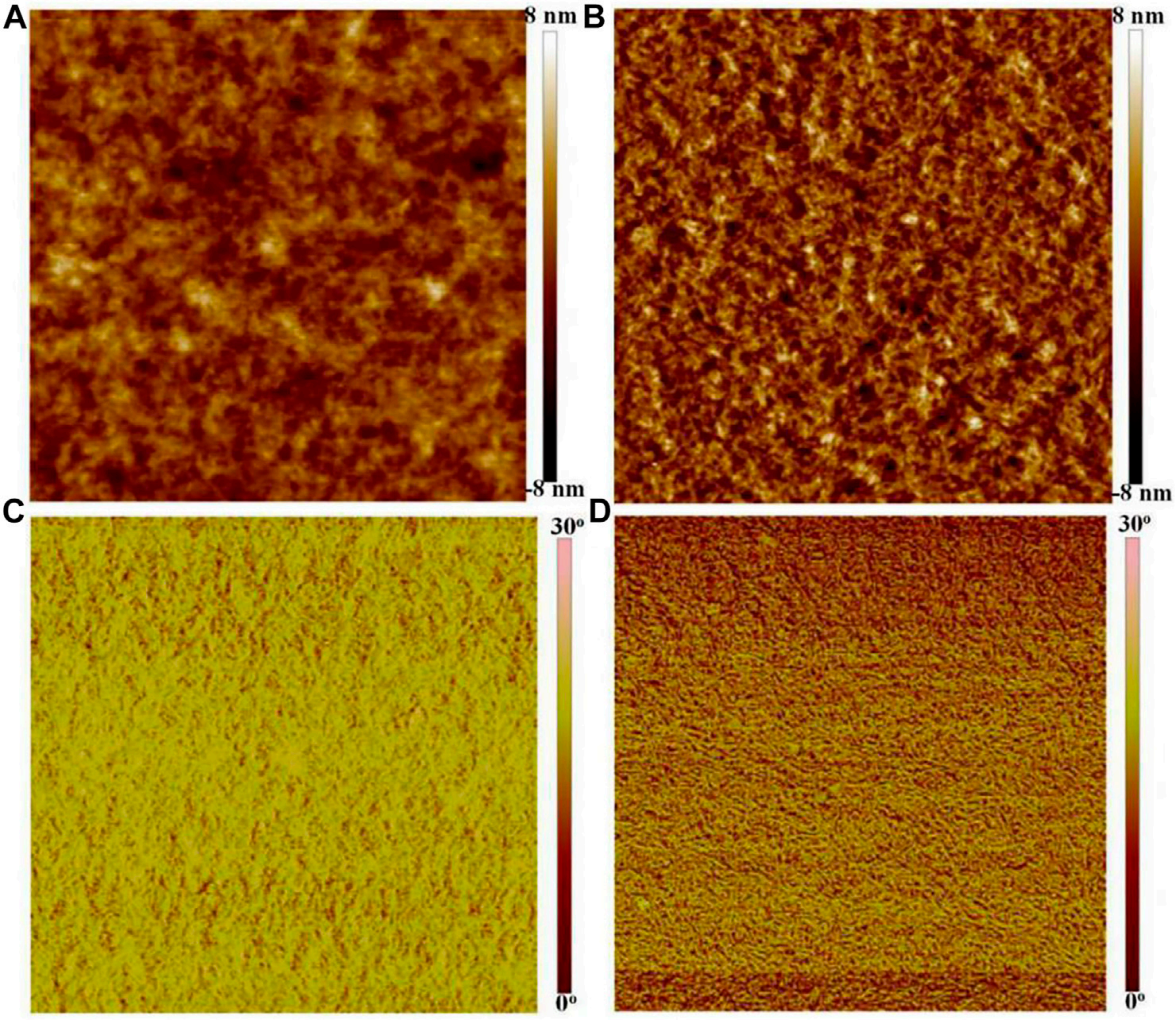
Height and phase images of the pristine films **(A,C)** and SOCl_2_-treated PEDOT:PSS films **(B,D)**. Scale bar, 2 μm × 2 μm.

Figure 3 shows the height and phase images of the PEDOT:PSS films through SPM. As shown in Figure 3A, the pristine PEDOT:PSS films with a root mean square (RMS) roughness of 1.32 nm exhibited an inferior phase-separated morphology. After the SOCl_2_ treatment, the PEDOT:PSS films with a higher RMS roughness of 1.95 nm showed the physically continuous and homogeneous networks that consisted of rich expanded/coiled chains for a free charge carrier hopping (Figure 3B). As shown in the phase image (Figure 3C), for the pristine films, the phases of the tip vibration are 16°−23° slower than that of the driving signals; whereas, for the SOCl_2_-treated PEDOT:PSS thin films, plenty of phases of the tip vibration are no more than 15° slower than that of the driving signals (Figure 3D), suggesting a homogeneous and smooth thin film. The results demonstrate a much better phase-segregated morphology of the PEDOT:PSS thin films induced by the SOCl_2_ treatment.

**FIGURE 4:**
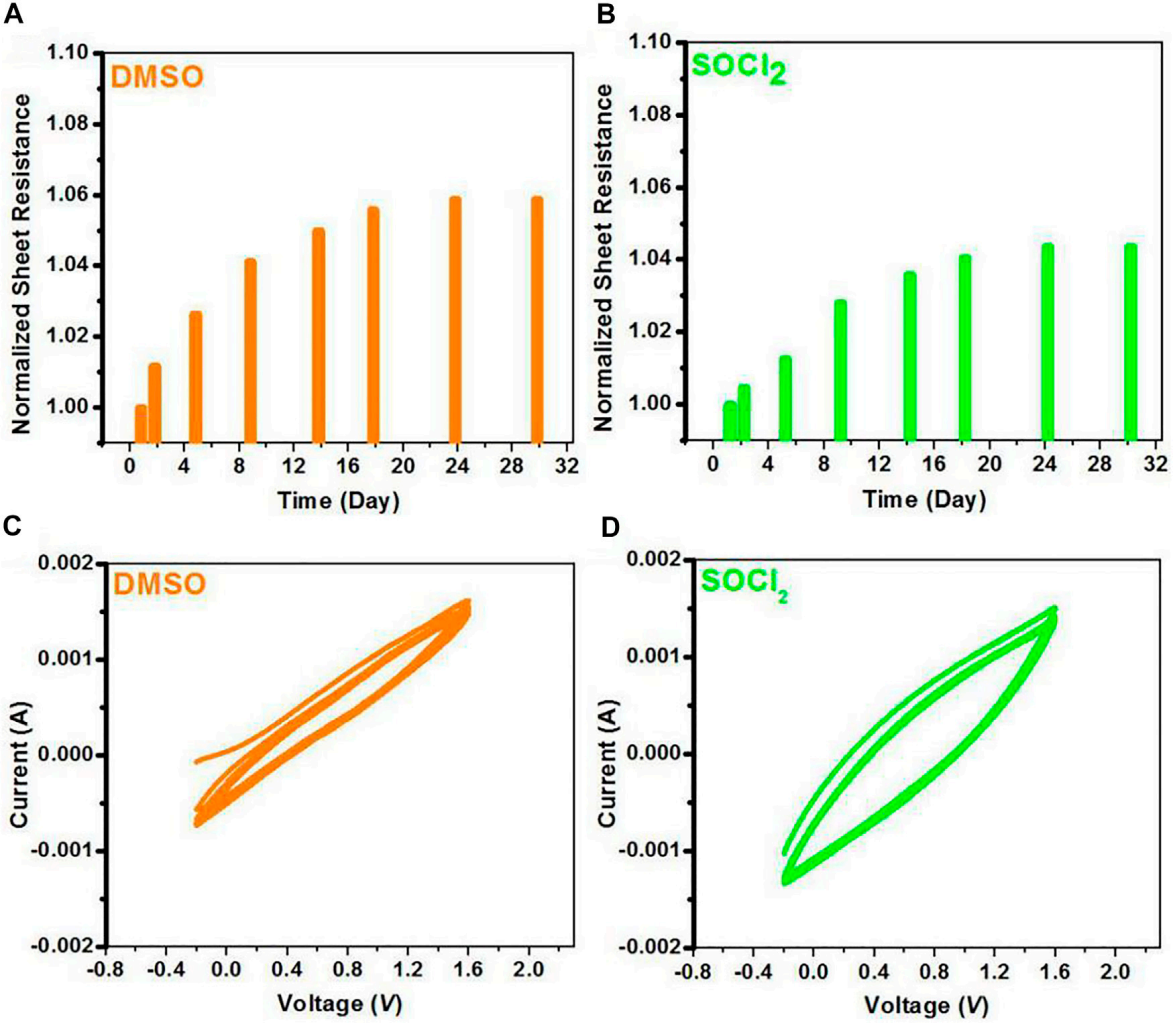
**(A,B)** Profiles of normalized sheet resistance of the DMSO- and SOCl_2_-treated PEDOT:PSS films exposed in ambient air. **(C,D)** CV curves of both films in anhydrous dichloromethane through using 0.1 M Bu_4_NPF_6_ as an electrolyte at a scan rate of 100 mV s^−1^.

PEDOT:PSS is prone to absorb water from the surrounding humid environments owing to the hygroscopic behavior of PSS and PSSH, thus potentially rendering the CPs vulnerable in ambient air. Volume expansion of PEDOT:PSS occurred in the ambient air would affect the charge hopping of the PEDOT:PSS films. Here, we investigated both electrical and electrochemical stabilities of the PEDOT:PSS films with 6 vol.% DMSO pre-treatments and the SOCl_2_ post-treatments. Note that both films were exposed in the ambient air (temperature: 20°C–26°C; relative humidity: 60%–70%). Figures 4A,B show the normalized sheet resistance of the PEDOT:PSS thin films treated by DMSO and SOCl_2_, respectively, in the ambient air for 30 days. An increase of 5.88% in *R*_sq_ was observed for the DMSO-treated PEDOT:PSS thin films, whereas an increase of 4.36% in *R*_sq_ was observed for the SOCl_2_-treated PEDOT:PSS films. The limited increase in square resistance indicates a better air stability of the SOCl_2_-treated PEDOT:PSS films. The promoted electrical stability of the SOCl_2_-treated PEDOT:PSS films was mostly attributed to 1) the less components of hydrophilic and insulating PSS components in PEDOT:PSS matrices and 2) the homogeneous and better phase-segregated morphology that potentially restrained the absorption of moisture and volume expansion.

Furthermore, the electrochemical behaviors of the PEDOT:PSS thin films were investigated through cyclic voltammetry (CV), as shown in Figures 4C,D. In the CV tests, the PEDOT:PSS thin films were spin-coated on ITO working electrodes in 0.1 M Bu_4_NPF_6_ (in CH_2_Cl_2_) solutions. The PEDOT:PSS electrodes with the SOCl_2_ treatment had the stable reversibility during the oxidation–reduction process for 10 cycles, probably due to the less PSS components and the improved electrical conductivity. We found that the charge capacity of the SOCl_2_-treated PEDOT:PSS thin films was larger than that of the DMSO-treated PEDOT:PSS thin films, suggesting more electron transferring in the doping and dedoping processes. The results demonstrate a better electrochemical stability of the PEDOT:PSS films achieved by the SOCl_2_ treatment.

### Photovoltaic Performances of Flexible OSCs

**FIGURE 5:**
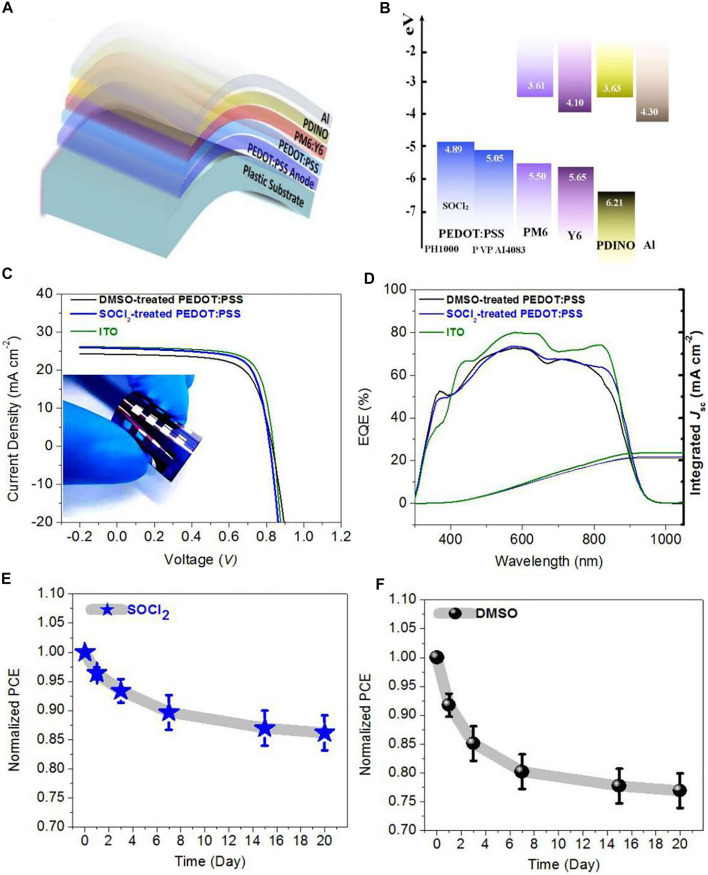
**(A)** Schematic diagram of flexible OSCs. **(B)** Energy levels of OSC components. **(C)** *J−V* characteristics of flexible OSCs with SOCl_2_- and DMSO-treated PEDOT:PSS electrodes and control OSCs fabricated on ITO/glass substrates. **(D)** EQE spectra of these OSCs. **(E,F)** Decays of normalized PCEs of the flexible OSCs with the PEDOT:PSS electrodes treated by SOCl_2_ **(E)** and DMSO **(F)**, respectively.

We fabricated the solution-processed flexible OSCs on the basis of the PEDOT:PSS transparent electrodes with the SOCl_2_ treatment. Figure 5A illustrates the flexible OSC structure, that is, PET (100 μm)/SOCl_2_-treated PEDOT:PSS (Clevios PH1000, 70 nm)/PEDOT:PSS (Clevios P VP AI4083, 25 nm)/PM6:Y6 (150 nm)/PDINO (10 nm)/Al (100 nm). The energy levels of the solar cell components are shown in Figure 5B. The good matching in energy levels among the SOCl_2_-treated PEDOT:PSS electrodes, PEDOT:PSS (P VP AI4083) HTLs, and PM6 allowed an efficient hole transfer into the PEDOT:PSS electrodes. Figure 5C shows the *J−V* characteristics of the three kinds of OSCs, including 1) flexible ones with the SOCl_2_-treated PEDOT:PSS electrodes, 2) flexible ones with the conventional 6 vol.% DMSO pre-treatments, and 3) rigid ones fabricated on ITO (110 nm)/glass substrates. It presented that the flexible OSCs with the SOCl_2_-treated PEDOT:PSS electrodes yielded an average PCE of 15.12% with a high fill factor (FF) of 0.719 and a short-circuit current density (*J*_SC_) of 25.73 mA cm^−2^ under the illumination of AM 1.5G, 100 mW cm^−2^. The PCE is higher than that (PCE:13.69%) of the control flexible OSCs with the DMSO-treated PEDOT:PSS electrodes that showed a *J*_SC_ of 24.21 mA cm^−2^ and a FF of 0.688. The higher PCE was mostly attributed to the higher electrical conductivity, suitable work function, and better phase-separated morphology of the SOCl_2_-treated PEDOT:PSS electrodes for an efficient charge collection. It is worth mentioning that the match (4.89, 5.05, and 5.50 eV) in energy levels of the SOCl_2_-treated electrodes, HTLs, and PM6 and the homogeneous morphology of the SOCl_2_-treated electrodes allow efficient hole transfer into the as-treated PEDOT:PSS electrodes for maximizing the PCE of the flexible OSCs. Furthermore, the PCE of the optimal flexible OSC_S_ is nearly comparable to that (PCE: 15.89%) of the control rigid OSCs fabricated on ITO (110 nm)/glass substrates that exhibited a high FF of 0.730 and a high *J*_SC_ of 26.06 mA cm^−2^. Table 1 summarizes the performance data of the flexible and rigid OSCs for reference.

**TABLE 1:** Photovoltaic parameters of flexible OSCs based on PEDOT:PSS electrodes and rigid OSCs based on ITO electrodes.

Transparent electrode	Treatment	*V*_OC_ [*V*]	*J*_SC_ [mA cm^−2^]	Fill factor	PCE [%]
PEDOT:PSS	SOCl_2_	0.818 (±0.005)	25.73(±0.22)	0.719 (±0.004)	15.12 (±0.20)
PEDOT:PSS	DMSO	0.821 (±0.005)	24.21(±0.24)	0.688 (±0.005)	13.69 (±0.26)
ITO	none	0.835 (±0.004)	26.06 (±0.19)	0.730 (±0.04)	15.89 (±0.08)

Figure 5D shows the EQE of the solution-processed flexible OSCs with the SOCl_2_- and DMSO-treated PEDOT:PSS electrodes and the control rigid OSCs fabricated on the ITO/glass substrates. The maximum EQE value of the optimal flexible OSCs with the SOCl_2_-treated PEDOT:PSS electrodes reached 84.6% at λ = 580 nm and 77.7% at λ = 700 nm, which demonstrated the high integrated *J*_SC_ of 25.11 mA cm^−2^. In contrast, for the control flexible OSCs with the DMSO-treated PEDOT:PSS electrodes, the EQE value at 580 nm reached 79.8% at λ = 580 nm and 73.8% at λ = 700 nm, which showed an integrated *J*_SC_ of 23.82 mA cm^−2^; whereas the control rigid OSCs fabricated on the ITO/glass showed the integrated *J*_SC_ as high as 25.70 mA cm^−2^. It can be seen that the EQE values are nearly consistent with the measured *J*_SC_ for all that flexible and rigid solar cells. The SOCl_2_ treatment raised the light utilization especially in the wavelengths of 500–900 nm. Therefore, the SOCl_2_-treated PEDOT:PSS thin films are suitable for efficient flexible OSCs fabrications as a flexible transparent electrode. We also investigated the stability of both flexible devices with SOCl_2_- and DMSO-treated PEDOT:PSS electrodes as a function of storage time. Figures 5E,F present the profiles of the normalized PCE of the flexible OSCs versus storage time. Obviously, the flexible OSCs with the SOCl_2_-treated PEDOT:PSS electrodes still maintain ∼86.2% of the initial PCE after the storage for 20 days in the glove boxes, whereas the flexible OSCs with the DMSO-treated PEDOT:PSS electrodes just held ∼76.9% of the initial PCE value. The improved device stability was attributed to the better electrochemical behaviors of the SOCl_2_-treated PEDOT:PSS electrodes and the raised electrical stability, as shown in Figure 4.

### Thermoelectric Performances of Flexible TEs

**FIGURE 6:**
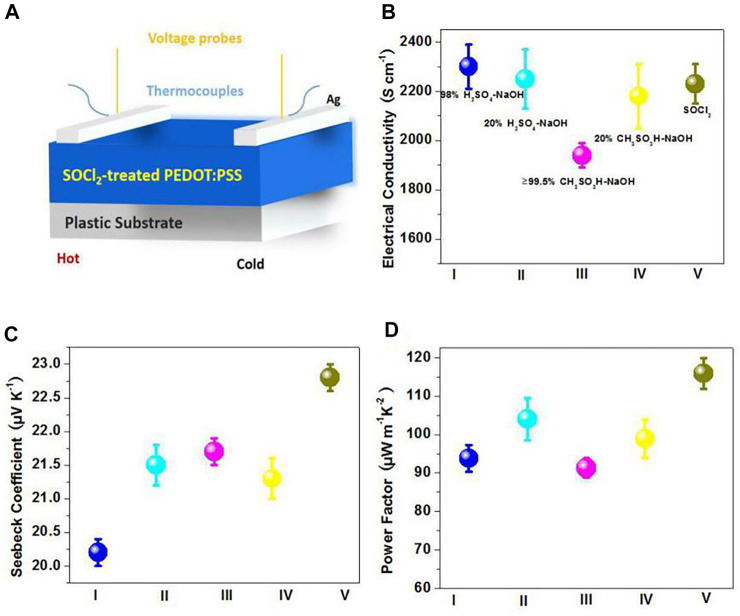
**(A)** Schematic diagram of flexible TEs. **(B–D)** Temperature-dependent electrical conductivity **(B)**, Seebeck coefficient **(C)**, and power factor (D) of the TEs. **I**: 98 wt.% H_2_SO_4_ (RT)-NaOH treatments, **II**: 20 wt.% H_2_SO_4_ (140°C)-NaOH treatments, **III**: ≥99.5 wt.% CH_3_SO_3_H (RT)-NaOH treatments, **IV**: 20 wt.% CH_3_SO_3_H (140°C)-NaOH treatments, and **V**: SOCl_2_ treatments.

Furthermore, to demonstrate the promising applications of the SOCl_2_-treated PEDOT:PSS thin films, we fabricated the flexible polymeric TEs on the basis of the PEDOT:PSS as active thermoelectric layers. The PEDOT:PSS TE device structure is illustrated in Figure 6A. Figures 6B–D show the temperature-dependent electrical conductivity, Seebeck coefficient, and PF of the PEDOT:PSS TEs, including 1) the flexible TEs based on the SOCl_2_-treated PEDOT:PSS films and 2) the four kinds of control rigid TEs based on the PEDOT:PSS films with the H_2_SO_4_ and sodium hydroxide (H_2_SO_4_-NaOH) multistep treatments and the CH_3_SO_3_H and NaOH (CH_3_SO_3_H-NaOH) multistep treatments. The conventional PEDOT:PSS films with strong acid treatments commonly showed a poor Seebeck coefficient and a limited PF (Fan et al., 2017). To increase the Seebeck coefficient and PF, the following NaOH base treatment was required to treat the acid-treated PEDOT:PSS films. As shown in Figure 6B, through using the SOCl_2_ treatment, the electrical conductivity of the flexible PEDOT:PSS thin films was enhanced from 0.3 to 2,230 S cm^−1^. The electrical conductivity of the flexible SOCl_2_-treated films is higher than that (1,940 S cm^−1^) of the ≥99.5 wt.% CH_3_SO_3_H-NaOH–treated films but slightly lower than that (2,300 S cm^−1^) of the 98 wt.% strong acid (i.e., H_2_SO_4_)–NaOH–treated films. Note that the H_2_SO_4_-treated PEDOT:PSS thin films were prepared on glass substrates rather than plastic underlying substrates, because of a large-domain damage of H_2_SO_4_ to most of plastic substrates; whereas the SOCl_2_ treatment is compatible with a flexible electrode solution manufacturing. The results demonstrate a better electrical conductivity of the SOCl_2_-treated PEDOT:PSS thin films. In terms of Seebeck coefficient, the PEDOT:PSS films with acid-NaOH treatment on glass substrates exhibited a Seebeck coefficient between 20.2 and 21.7 μV K^−1^; whereas the SOCl_2_ treatment at room temperature induced a considerably high Seebeck coefficient of 22.8 μV K^−1^. As a result, the SOCl_2_-treated PEDOT:PSS flexible thin films yielded the best PF value of 115.9 μW m^−1^ K^−2^, which outperformed these control rigid PEDOT:PSS TEs that had a relatively low PF of 91.3 μW m^−1^ K^−2^ for ≥99.5 wt.% CH_3_SO_3_H (RT)-NaOH treatments; 93.8 μW m^−1^ K^−2^ for 98 wt.% H_2_SO_4_ (RT)-NaOH treatments; 98.9 μW m^−1^ K^−2^ for 20 wt.% CH_3_SO_3_H-NaOH treatments; and 104.0 μW m^−1^ K^−2^ for 20 wt.% H_2_SO_4_-NaOH treatments, respectively. Note that the diluted acid treatments of 20 wt.% CH_3_SO_3_H and 20 wt.% H_2_SO_4_ were conducted at a higher temperature of 140°C. The results demonstrate that the simple SOCl_2_ treatment is superior to these multistep acid-NaOH treatments in terms of raising Seebeck coefficient and PF.

The mechanism for the TE property enhancement of the PEDOT:PSS films treated by SOCl_2_ may be explored as below. In our recent works, the conventional CF_3_SO_3_H (Wan et al., 2021) and HClO_4_ (Wan et al., 2020a) treatments commonly enabled a doped PEDOT:PSS film, leading to a blue shift of the Raman peak at 1,430–1,440 cm^−1^. When the pristine PEDOT:PSS film was subject to the SOCl_2_ treatment, a blue shift of the Raman peak at 1,430–1,440 cm^−1^ was observed here, suggesting a dedoping process of PEDOTs and a localization of the positive charges on PEDOTs. Many protons would be removed from the PEDOTs during the SOCl_2_ processing, resulting in a lower charge concentration and thus enhancing the Seebeck coefficient of the PEDOT:PSS thin films. In recent literature, a rigid PEDOT:PSS/glass sample with optimized H_2_SO_4_ soaking treatments and optimized base treatments exhibited a record-high PF of 334 µW m^−1^ K^−2^ due to an inspiring Seebeck coefficient. The PF of our flexible PEDOT:PSS thin films lagged behind the record-high PF of the rigid and opaque PEDOT:PSS/glass samples that required a precise optimization of H_2_SO_4_ soaking treatments and base treatments. However, the H_2_SO_4_ treatments caused a large-domain damage to most of flexible plastic substrates, thereby unsuitable to prepare flexible PEDOT:PSS TEs; meanwhile, the following NaOH base treatments changed the color of the PEDOT:PSS films from light blue to dark blue, therefore, hampering the realization of highly transparent TE devices. In this work, the flexible TEs based on the SOCl_2_-treated PEDOT:PSS thin films not only have the advantages of simple solution-treatment/manufacturing, good flexibility, and highly optical transmittance but also are desirable to realize the kinds of flexible devices, such as flexible and transparent TEs, flexible OSCs, and integrated electronics involving flexible TEs and OSCs.

## Conclusion

In summary, we demonstrated the high-performance flexible OSCs and flexible TEs on the basis of the SOCl_2_-treated PEDOT:PSS electrodes. The SOCl_2_ treatment enabled a homogeneous and smooth PEDOT:PSS electrode with highly electrical conductivity of 2,230 S cm^−1^ and Seebeck coefficient of 22.8 μV K^−1^. The resultant flexible OSCs yielded the high PCE of 15.12%. It is almost attributed to the SOCl_2_ treatment that endowed the PEDOT:PSS electrodes with highly optical and electrical characteristics, homogeneous and smooth morphology, and high work function (4.89 eV). Besides, the flexible PEDOT:PSS TEs not only had the merit of high transmittance but also yielded a PF of 115.9 μW m^−1^ K^−2^, which was higher than that (91.3–104.0 μW m^−1^ K^−2^) of the four kinds of rigid PEDOT:PSS TEs with acid and NaOH multistep treatments. The work opens a venue to realize more efficient solution-processed flexible OSCs and flexible TEs that convert both luminous and thermal energies to electricity.

## Data Availability

The original contributions presented in the study are included in the article/supplementary material. Further inquiries can be directed to the corresponding authors.
